# Effect of an Ergonomics Educational Program on Musculoskeletal Disorders in Nursing Staff Working in the Operating Room: A Quasi-Randomized Controlled Clinical Trial

**DOI:** 10.3390/ijerph17197333

**Published:** 2020-10-08

**Authors:** Tahereh Abdollahi, Shadan Pedram Razi, Daryoush Pahlevan, Mir Saeed Yekaninejad, Sara Amaniyan, Christina Leibold Sieloff, Mojtaba Vaismoradi

**Affiliations:** 1 School of Nursing and Midwifery, Tehran University of Medical Sciences, Tehran 1419733171, Iran; taherehabdollahi2@gmail.com (T.A.); pedramrazi@tums.ac.ir (S.P.R.); 2 Social Determinants of Health Research Centre, Semnan University of Medical Sciences, Semnan 3514799422, Iran; daryoushpahlevan@gmail.com; 3 Department of Epidemiology and Biostatistics, School of Public Health, Tehran University of Medical Sciences, Tehran 1417613151, Iran; yekaninejad@sina.tums.ac.ir; 4 Student Research Center, Semnan University of Medical Sciences, Semnan 3514799422, Iran; s-amaniyan@razi.tums.ac.ir or; 5 College of Nursing, Montana State University, Bozeman, MT 172220, USA; csieloff@montana.edu; 6 Faculty of Nursing and Health Sciences, Nord University, 8049 Bodø, Norway

**Keywords:** ergonomics, educational program, musculoskeletal disorders, occupational health, operating room nurse

## Abstract

*Background:* Nursing staff working in the operating room are exposed to risk factors that can cause musculoskeletal disorders (MSDs) and work-related disabilities. The use of ergonomics principles can help with the prevention of MSDs. This study aimed to examine the effect of an ergonomics educational program on MSDs among nursing staff working in the operating room. *Methods:* In this pragmatic parallel group quasi-randomized controlled clinical trial, 74 nursing staff working in the operating rooms of two teaching hospitals participated. The hospitals were randomly assigned to either the intervention or the control group and all nursing staff working in the operating room of each hospital were invited to take part in this research. They were initially assessed for the prevalence and risk of MSDs by using the Nordic questionnaire and the rapid entire body assessment (REBA) checklist. The intervention group received the ergonomics educational program and were assessed in two-week intervals over a period of three months. At the end of the study, the risk and prevalence of MSDs were compared between the intervention and control groups. *Results:* Statistically significant differences were reported between the groups in terms of the prevalence and risk of MSDs. The overall risk of MSDs decreased in the intervention group after the educational program (*p* = 0.03). The reduction in the prevalence of MSDs in the different parts of the body in the intervention group was as follows: ankle (*p* = 0.005), hand/wrist (*p* = 0.041), low back (*p* = 0.000), the neck (*p* = 0.003), hip (*p* = 0.001) and shoulder (*p* = 0.043). *Conclusion:* The education of nursing staff about ergonomics can influence the prevalence and risk of MSDs. Therefore, it should be incorporated into the degree education and on-the-job training initiatives for nurses working in the operating theatre in order to reduce workplace injuries and associated absences, and increase the quality of care delivered by them. This clinical trial has been registered in the Iranian Registry of Clinical Trials: IRCT2015081823677N1.

## 1. Introduction

Nurses are exposed to risks of work-related injuries leading to burnout and reductions in quality of care [1,2,3]. These injuries can involve all body organs and particularly the musculoskeletal system [4,5]. 

Musculoskeletal disorders (MSDs) are defined as dysfunctions affecting various parts of the body, including muscles, bones, joints, and spinal discs [6]. These dysfunctions are mainly caused by repetitive manual labor, lifting heavy loads, prolonged standing, and working in fixed or inappropriate postures [7,8]. Work-related MSDs are characterized by decreased strength or range of motion, pain and swelling [9]. 

The prevalence of occupational injuries and work-related pressures among nursing staff working in the operating room is higher than in nonspecialized nurses, intensive care nurses, and radiologic technicians [10]. Long-term standing, static body posture, using tools such as retractors during surgical procedures, and manual tasks, such as pulling, pushing, and lifting surgery sets and patients, can cause discomfort and disorders in the musculoskeletal system [11]. However, occupation-specific interventions and rehabilitation options have the potential to reduce functional disabilities among nursing staff that may result from these injuries [12]. 

### 1.1. Significance of Ergonomics in Nursing 

The risk of developing MSDs can be predicted based on individual, psychosocial and ergonomic factors [13]. Ergonomics as a science discusses how to fit a job to an individual’s physical and psychological characteristics in such a way as to prevent any harm to the individual’s efficiency and wellbeing [9]. The knowledge and application of ergonomics can prevent the onset and progress of MSDs and improve health status [14]. Working in appropriate ergonomics conditions can increase nurses’ motivation and job satisfaction, and reduce their job stress, absenteeism, occupational diseases, and work-related accidents [15]. A study by Szeto et al. (2013) showed that the multifaceted ergonomic intervention program had many advantages and promoted occupational health among community nurses [16]. According to Moazzami et al., (2016) ergonomics educational interventions based on the transtheoretical model can facilitate the creation of changes in body movements among nurses, from the contemplation and preparation stages to the action stage for adopting a correct body posture in the operating theatre [8]. A study on nurses working in general wards showed a reduction in musculoskeletal pain in the areas of the neck, shoulder and knee after the implementation of an ergonomic educational intervention [17]. However, some studies have reported no significant benefit or the limited success of ergonomics interventions on MSDs [18,19], which can be attributed to variations in their educational interventions, samples and settings, data collection tools, and the research structure. Therefore, more studies should be carried out to develop conclusive evidence on the effectiveness of ergonomics educational interventions in the reduction of MSDs among clinical nurses. Such studies can help with the development of new work techniques for nursing staff working in the operating room, and their incorporation into daily care routines that further reduce work-related injuries [20]. A successful ergonomics program designed to prevent MSDs among nursing staff working in the operating room should create a clear understanding of their roles and responsibilities, and should explain ergonomics risk factors in such a high-risk environment [21].

### 1.2. Education in Ergonomics for Nurses

Education and training about ergonomics has been suggested for the reduction of MSDs among nursing staff [22,23]. However, ergonomics is not routinely taught during undergraduate nursing education and on-the-job training given to nurses [24]. A lack of knowledge about ergonomics not only leads to disturbances in the workflow, but also increases physical impairments and potential hazards for the healthcare team in the operating room [20]. Operating room nurses deal with a very exhausting, busy, and overloading work environment, which requires more effort, organization and training [25]. Therefore, appropriate interventions and solutions should be identified, evaluated and applied in order to reduce ergonomic risk factors and MSDs among this important group in the healthcare team. Therefore, this study aimed to examine the effect of an ergonomics educational program on MSDs among nursing staff working in the operating room.

The research hypothesis was as follows:

**Hypothesis** **1.** The ergonomics educational program can reduce the prevalence and risk of MSDs among nursing staff working in the operating room.

## 2. Materials and Methods 

### 2.1. Design

A pragmatic parallel group quasi-randomized controlled clinical trial with a pre-test–post-test design was conducted from April to July 2018 (Supplementary file: CONSORT 2010 checklist). This clinical trial has been registered on the Iranian Registry of Clinical Trials: IRCT2015081823677N1. The trial protocol can be accessed via https://www.irct.ir/trial/20188.

### 2.2. Setting and Participants 

The subjects were selected from nursing staff consisting of nurses (bachelor’s degree in nursing) and nurse assistants (associate degree in nursing) working in the operating rooms of two teaching hospitals in an urban area of Iran. Assuming the probability of data contamination between intervention and control groups, two hospitals were selected using a convenience method. Given the homogenous conditions of the hospitals in terms of the staffing pattern, type of surgery, and workflow and workload, one hospital was randomly chosen as the control group and another hospital as the intervention group through coin flipping by the first author (TA). Next, all nursing staff working in the operating rooms were enrolled into the study using the convenience sampling method. It is noted that the identity of the research project hindered the blinding of the participants, but the data analyst (MSY) was not informed of the group assignments to prevent bias in the data analysis process. 

### 2.3. Sampling and Eligibility Criteria 

The sample size was estimated using a statistical power analysis and the following formula, whereby statistical significant was set at α = 0.05, power = 0.90, there was a 10% possibility of dropout, and the expected difference between the groups at the two measurement times was 2:

(1) $$n=\frac{{\left(z_{1-\frac{\alpha }{2}}+z_{1-\beta }\right)}^{2}\times 2\sigma^{2}}{\Delta^{2}}$$

As a result, 33 persons were required for recruitment in each group, but all nursing staff (*n* = 79) working in the operating rooms were recruited into this study to reduce the possibility of contamination. However, according to the following exclusion criteria, five subjects were excluded, and therefore this research was continued with 37 nurses in each group. We excluded those subjects who had a medical history of MSDs (*n* = 2), and those who had a very low risk level of MSDs in terms of working part time (*n* = 1) and having less than one year of work experience (*n* = 2). 

**Figure 1:**
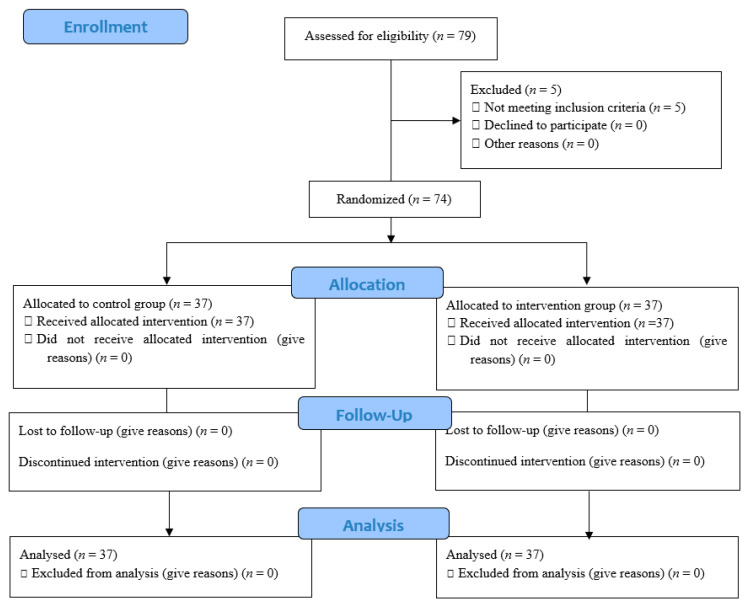
The process of the study according to the CONSORT flow diagram.

A summary of the study process based on the CONSORT flow diagram (2010) has been presented in Figure 1.

### 2.4. Outcomes and Data Collection

The main research outcomes of this research were the prevalence and the risk of MSDs, which were assessed using the following data collection tools:

⚬ The modified version of the standardized Nordic questionnaire. This is a general questionnaire aided by a body map to indicate nine symptom sites of the neck, shoulders, upper back, elbows, low back, wrist/hands, hips/thighs, knees and ankles/feet for the assessment of the prevalence of MSDs in the last 12 months and last 7 days, which have prevented normal activities [26,27];
⚬ The rapid entire body assessment (REBA) checklist for the assessment of the risk of MSDs [28]. Bias might have occurred during the completion of the REBA checklist through the researchers’ direct observation of the subjects’ activities. Therefore, the subjects’ postures and activities during surgery, working with the patient, packaging and sterilizing equipment, recovery and computer work were video-recorded by the researcher, after obtaining appropriate ethical permission. The films were then used to recheck activities and reduce the number of observational failures on the part of the first researcher (TA). Subsequently, the most frequent physical conditions and postures of the subjects during the 20 min observation period were scored. To recheck the videos and eliminate the possibility of bias, the videos were then reviewed and confirmed by a colleague who was a master of ergonomics and was not a member of the research group, so as to enhance the accuracy of the completed checklist. The overall agreement between evaluators for the REBA checklist was calculated as 0.82 using Cohen’s Kappa measures.

Furthermore, the demographic data questionnaire with questions about the subjects’ age, gender, education level, job history, job title and job task were used. Moreover, the nurses’ job satisfaction was assessed using the Minnesota Job Satisfaction Questionnaire (MJSQ). This self-reporting questionnaire includes 19 questions with a 5-point Likert scale. A lower score indicates a lower level of job satisfaction [29].

These questionnaires were assessed in terms of face and content validities, and reliability, to ensure their suitability for data collection. Before the intervention, both groups completed the demographic data questionnaire, the Nordic questionnaire, the REBA checklist and the MJSQ in the presence of the first researcher (TA), which lasted about 20 min. The results of the REBA checklist and Nordic questionnaire were extracted to identify the ergonomic educational needs of the subjects. Both groups were assessed again using these questionnaires. After that the intervention was finished.

### 2.5. Intervention

In collaboration with an occupational medicine specialist, an educational program on ergonomics for the nursing staff working in the operating room was developed. Following a thorough literature review, the educational program was prepared focusing on the ergonomic risk factors and practical methods for removing risk factors or controlling them within the operating room environment. 

The subjects in the intervention group attended the two-hour educational session about the principles of ergonomics, presented by the occupational medicine specialist, via lectures and slide presentations. To enhance the quality of education and increase attendees’ participation, this educational session was held in three small groups consisting of 12–13 individuals over three consecutive days, each day for one group. 

The educational sessions covered topics such as (i) the definition, purposes and principles of ergonomics, (ii) occupational stress, (iii) MSDs, (iv) prevalence of MSDs among nursing staff, (v) factors contributing to the development of MSDs, and (vi) methods used to prevent them. The sessions were held in a classroom next to the operating room, and the first author (TA) was present during the educational sessions. In addition, an educational pamphlet, summarizing the educational materials, was given to each subject at the end of the session, and subjects were encouraged to address their uncertainties through asking questions. 

The subjects were monitored over a period of three months. Since the majority of ergonomic changes required at least two weeks to be evaluated, the first author (TA) assessed the subjects every two weeks to determine their educational needs and provide individual and face-to-face education, with an emphasis on the materials originally provided during the educational sessions. 

The subjects completed the Nordic questionnaire every two weeks, and the first author (TA) video-recorded their activities. The subjects were asked to watch the movies taken of them during the data collection, and were provided with details about their weaknesses and strengths, in terms of ergonomics, during their practice in the operating room. They were also reminded to study the educational pamphlet and ask their questions.

The subjects in the control group did not receive any education and were only assessed using the data collection tools at the beginning and end of the study. In the third month of the intervention, both groups were reassessed using the questionnaires. 

### 2.6. Ethical Considerations

Institutional Review Board approval for the study was granted by the Ethics Committee affiliated with the university with which the second author (SPR) was affiliated (decree code: 9111196041-1). Furthermore, permission to use the data collection questionnaires was obtained from the developer of each questionnaire. 

The first researcher (TA) visited the operating rooms of the two hospitals and described the aim and procedure of the study to the operating room nursing staff. The purposes and procedures of the study were explained to the potential subjects and written informed consent was signed by each subject prior to participation. They were informed of their rights to terminate their participation at any time throughout the study process without being penalized. In addition, they could request to receive a report of the study findings. Finally, permission to video-record the activities and postures of the subjects during their regular work was obtained. They were also assured that their names would remain confidential, and the collected data would be used only for the purpose of research. 

### 2.7. Data Analysis

The SPSS software for Windows version 21 was used for statistical analysis (IBM SPSS Inc., Chicago, IL, USA) for the research data. Descriptive statistics consisting of mean, standard deviation, number and percentage were used. Furthermore, inferential statistical tests included the chi-squared test and t-test for the assessment of relationships between demographic variables; the ANOVA and ANCOVA test, and logistic regression to compare and identify relationships between the research variables. The significance level (*p*) was set at <0.05.

## 3. Results

### 3.1. Demographic Data of the Participants

After the exclusion of five nurses based on the exclusion criteria, each intervention and control group consisted of 37 nurses working in the operating rooms of two hospitals. The mean (SD) ages of the subjects in the control and the intervention groups were 26.64 (SD = 5.83) and 31.45 (SD = 8.19) years, respectively. The mean of work experience was 2.70 years (SD = 5.07) in the control group and 8.51 years (SD = 7.64) in the intervention group. Statistically significant differences were reported between the ages and work experiences of the subjects in the groups. Therefore, the groups were not homogeneous in terms of age and work experiences (Table 1). 

However, no statistically significant difference was reported between the groups in terms of other demographic characteristics, including gender, job tasks, education level and work shift. So, the groups were homogeneous (Table 2). 

### 3.2. Prevalence of MSDs

Lower back pain had the highest prevalence in both groups before the intervention (control = 62.2%, intervention = 54.1%, *p* = 0.48). Before the intervention, no statistically significant differences were reported between the groups in terms of the prevalence of MSDs in the different parts of the body except for hip (intervention = 24.3%, control = 8.1%, *p* = 0.058). As such, the groups were homogeneous in terms of the prevalence of MSDs before the intervention (Table 3). 

After the intervention, statistically significant differences were observed between the groups in terms of the prevalence of MSDs in the different parts of the body, as follows: ankle (*p* = 0.005), hand wrist (*p* = 0.041), low back (*p* =0.000), the neck (*p* = 0.003), hip (*p* = 0.001) and shoulder (*p* =0.043). Consequently, the prevalence of MSDs was significantly lower in the intervention group compared with the control group. No significant statistical differences were reported between the control and the intervention groups with regards to the prevalence of MSDs in the elbow (control = 8.1%, intervention = 5.4%, *p* = 0.640), back (control = 37.8%, intervention = 21.6%, *p* = 0.126) and knee (control = 54.1%, intervention = 37.8%, *p* =0.161) (Table 3). 

The effects of the demographic variables, such as primary pain level, age, work experience and work shift, on the research variables were removed through the logistic regression analysis. Accordingly, the primary REBA score was influenced by the effect of the intervention, and the prevalence of MSDs was significantly lower in the intervention group compared with the control group (Table 4), as follows: ankle (*p* = 0.007), hand/wrist (*p* = 0.038), back pain (*p* = 0.000), the neck (*p* = 0.006), hip (*p* = 0.001) and shoulder (*p* =0.012).

### 3.3. Risk of MSDs

No statistically significant difference was reported between the groups in terms of the pre-study risk of MSDs (Table 5). However, after the intervention, a statistically significant difference was reported between the groups in terms of the post-study risk of MSDs. In the intervention group, before the educational program, 13.5% were at low risk of developing MSDs, 51.4% were at medium risk, and 35.1% were at high risk. After the intervention, the high risk of developing MSDs reduced to 0%. Furthermore, they mostly (48.6%) had a low risk level, indicating the effectiveness of the ergonomics educational program (*p* = 0.03).

### 3.4. Job Satisfaction

No statistically significant difference between the control group (mean = 50.17, SD = 11.75) and the intervention group (mean = 45.05, SD = 12.63) in terms of job satisfaction after the educational program was reported (Table 6).

## 4. Discussion

This research examined the effect of the ergonomics educational program on the prevalence and risk of MSDs among nursing staff working in operating rooms. Accordingly, significant differences between the control and intervention groups, in terms of the prevalence and risk of MSDs, were reported. Despite the heavy workload and the high prevalence of MSDs (88% in at least one body region in a one-year period) among Iranian nurses [30], no appropriate education about ergonomics is provided to them. 

In our study, lower back pain was identified as the most common MSD among the nursing staff working in the operating room. Generally, work-related MSDs influence soft tissues in areas of the neck, back, shoulder, elbow, hand, wrist and fingers [31]. Consistent with our research findings, Davis and Kotowski (2015) showed that the prevalence of MSDs among nurses and nurse assistants was the highest in the low back rather than shoulders and the neck [32]. Similarly, Zayed et al.’s (2019) study found that the lower back was the most affected site (56.5%), followed by the neck (51.5), and the least affected site was the elbow (18.5%) [33]. Standing for long hours during surgical operations, fixed postures, back hyperextension or flexion, moving, pushing and lowering heavy loads such as trays, monitors and patients, and transfers of the patients from beds to stretchers or vice versa are the main causes of MSDs among operating room staff [15].

Before the educational program, the subjects in our study were mostly at medium risk of developing MSDs. Tirgar et al. (2013), in a study on surgeons, reported a REBA score between four and seven, indicating a medium risk level and a need for interventions to reduce related occupational hazards [34]. Another study by Ratzon et al. (2016) on hospital nurses reported an improvement in the REBA score and in nurses’ postures that was considered a risk factor for work-related musculoskeletal discomfort, but observed no significant differences in the number of body parts in pain or in the level of musculoskeletal pain [35].

After the intervention, the results of our study demonstrated a statistically significant relationship between the education program related to ergonomics and the risk levels of MSDs. The percentage of the subjects who were at a medium risk underwent a minor change, but the percentage of those at a high-risk level reached zero, and an increase was observed in the percentage of individuals at a low risk level. These results demonstrated that the education intervention acted to reduce the risk levels of MSDs in operating room nursing staff. Similarly, Mahmud et al. (2011) indicated that ergonomics education reduced MSDs among office workers during a six-month follow up [13]. As mentioned earlier, the REBA checklist’s items assessed individuals’ physical condition, pressure on their limbs, and the type of activity such as repetitive work. Since the related mean score of the REBA declined in this study, the subjects experienced a reduction in their risk of MSDs. Therefore, the education intervention assisted them to change their physical condition at work, adopt proposed solutions to reduce their risk of MSDs, and avoid pressure and repetitive work. Nevertheless, since the etiology of MSDs is multifactorial with the involvement of physical and psychosocial factors related to the work environment, preventive strategies should consider all aspects of the work environment [31].

In this study, no statistically significant relationship was found between the education of ergonomics and the subjects’ job satisfaction. Various factors consisting of individual and organizational factors, such as the nature of the task, professional status, relationships in the workplace, being supported by managers, autonomy and decision-making, can influence nurses’ job satisfaction [36,37]. It is possible that, in addition to education regarding ergonomics, the above-mentioned factors would have influenced the nurses’ job satisfaction, which has not been considered in this study. As this is the first study to examine the relationship between job satisfaction and education of ergonomics, it is recommended that future studies further examine such a relationship. 

Given the research feasibility factors, the sample size of the nursing staff working in the operating room was small. Further, our selected subjects were not completely comparable in terms of working years, work schedule/shift and other unidentified factors that might have affected the odds of suffering from MSDs. In addition, this research assessed the effect of the intervention over a three-month period. Therefore, more studies should be conducted with the consideration of all confounding factors affecting the prevalence and risk of MSDs among nurses working in the operating room, and with a longer intervention duration to generate more robust evidence concerning the effectiveness of our educational program.

## 5. Conclusions

As demonstrated by this study, the prevalence of MSDs in the intervention group was significantly lower than that in the control group after the ergonomics educational program. Furthermore, when compared to the control group, the level of risk for MSDs decreased significantly in the intervention group. Therefore, the findings of this study support the conclusion that education regarding ergonomics may affect the prevalence and risk level of MSDs among nursing staff working in the operating room. The incorporation of ergonomics education into degree education and on-the-job training initiatives for nurses working in the operating theatre may help with the further improvement of the workplace environment, and consequently reduce workplace injuries and associated absences, and increase the quality of care delivered by them. 

It is suggested to replicate this research, and to recruit nursing staff working in general nursing wards and the community healthcare settings, as well as family caregivers that provide care to patients with long-term diseases in their home, in order to assess the generalizability of our findings and the adoption of our educational program in other caring settings. 

## Figures and Tables

**Table 1:** Mean age and work experience of the subjects in the intervention and control groups.

Group	Control		Intervention		*p*-Value
	Mean	SD	Mean	SD	
Age (years)	26.64	5.83	31.45	8.19	0.005
Work experience (years)	2.70	5.07	8.51	7.64	0.000

SD: Standard deviation.

**Table 2:** Other demographic characteristics of the subjects in the groups.

		Control (*n*%)		Intervention (*n*%)		*p*-Value
Gender	Male	7	18.9%	11	29.7%	0.278
	Female	30	81.1%	26	75.3%	
Education level	Associate degree	5	13.5%	12	32.4%	0.053
	Bachelor’s degree	32	86.5%	25	67.6%	
Work shift	Morning	22	59.5%	5	13.5%	0.000
	Circulating	15	40.5%	32	86.5%	
Job task	Scrub or circulate	25	67.6%	19	51.4%	0.225
	Anesthesia	10	27%	12	32.4%	
	Others	2	5.4%	6	16.2%	
Total		37	100	37	100	

**Table 3:** The prevalence of pain before and after the intervention in the groups.

Pain	Control		Intervention			
	*n*%		*n*%		*p*-Value	
	Before	After	Before	After	Before	After
Neck	17 (45.9)	12 (32.4)	16 (43.2)	2 (5.4)	0.810	0.003
Shoulder	13 (35.1)	14 (37.8)	14 (37.8)	5 (13.5)	0.892	0.043
Elbow	2 (5.4)	3 (8.1)	2 (5.4)	2 (5.4)	1.000	0.640
Hand wrist	9 (24.3)	12 (32.4)	9 (24.3)	5 (13.5)	1.000	0.041
Back	16 (43.2)	14 (37.8)	16 (43.2)	8 (21.6)	1.000	0.126
Low back	23 (62.2)	24 (64.9)	20 (54.1)	8 (21.6)	0.480	0.000
Hip	9 (24.3)	12 (32.4)	3 (8.1)	1 (2.7)	0.058	0.001
Knee	21 (56.8)	20 (54.1)	17 (45.9)	14 (37.8)	0.351	0.161
Ankle	13 (35.1)	16 (43.2)	11 (29.7)	5 (13.5)	0.610	0.005

**Table 4:** The logistic regression analysis of pain in different parts of the body after the intervention and the results of the rapid entire body assessment (REBA) for assessing the risk of musculoskeletal disorders (MSDs).

Logistic Regression	Variables	OR	95%CI	*p*-Value
**Regression analysis of pain in different parts of the body after the intervention**	Intervention vs. control	0.10	0.02–0.51	0.006
	Primary neck pain (referent = no)	7.80	1.78–34.27	0.007
	Intervention vs. control	0.20	0.05–0.69	0.012
	Primary shoulder pain (referent = no)	4.75	1.43–15.71	0.0011
	Intervention vs. control	0.36	0.11–1.18	0.093
	Primary back pain (referent = no)	9.28	2.11–31.12	0.000
	Intervention vs. control	0.12	0.04–0.39	0.000
	Primary lumbar pain (referent = no)	5.81	1.77–19.01	0.004
	Intervention vs. control	0.46	0.03–5.80	0.552
	Primary elbow pain (referent = no)	11.69	0.78–173–88	0.074
	Intervention vs. control	0.25	0.70–0.92	0.038
	Primary hand wrist pain (referent= no)	6.45	1.81–22.92	0.004
	Intervention vs. control	0.03	0.002–0.56	0.001
	Primary hip pain (referent = no)	64.50	6.66–624.54	0.000
	Intervention vs. control	0.53	0.16–1.74	0.291
	Primary knee pain (referent = no)	19.84	5.87–67.07	0.000
	Intervention vs. control	0.16	0.04–0.60	0.007
	Primary ankle pain (referent= no)	7.41	2.17–25.30	0.001
**The results of the REBA for assessing the risk of MSDs**	Intervention vs. control	0.22	0.07–0.70	0.011
	Mean of the REBA scores (referent = no)	11.67	2.47–55.12	0.002

**Table 5:** The risk of developing MSDs before and after the intervention in the groups.

Risk	Low		Medium		High		*p*-Value	
	*n*%		*n*%		*n*%			
Time	Before	After	Before	After	Before	After	Before	After
Intervention group	5 (13.5%)	18 (48.6%)	19 (51.4%)	19 (51.4%)	13 (35.1%)	0 (0%)	0.35	0.03
Control group	8 (21.6%)	10 (27%)	25 (67.6%)	26 (70.3%)	4 (10.8%)	1 (2.7%)		
Total	13 (17.5%)	28 (37.8%)	44 (59.5%)	45 (60.8)	17 (23%)	1 (1.4%)		

MSDs: musculoskeletal disorders.

**Table 6:** The comparison of job satisfaction between the groups before and after the intervention.

Time/Group	Before		After		Statistical Analysis ANCOVA Test *, *p* Value
	Mean	SD	Mean	SD	
Intervention	48.07	10.45	45.05	12.63	df = 1, F = 2.90, 0.093
Control	50.28	12.70	50.17	11.75	

* Adjusted for the age and work experience variables.

## Supplementary Materials

The following are available online at https://www.mdpi.com/1660-4601/17/19/7333/s1, supplementary file: CONSORT 2010 checklist.
